# Aptamer-Functionalized Hybrid Nanostructures for Sensing, Drug Delivery, Catalysis and Mechanical Applications

**DOI:** 10.3390/ijms22041803

**Published:** 2021-02-11

**Authors:** Margarita Vázquez-González, Itamar Willner

**Affiliations:** Center for Nanoscience and Nanotechnology, Institute of Chemistry, The Hebrew University of Jerusalem, Jerusalem 91904, Israel

**Keywords:** nanotechnology, nanoparticles, microcapsules, graphene oxide, DNA origami, DNAzymes

## Abstract

Sequence-specific nucleic acids exhibiting selective recognition properties towards low-molecular-weight substrates and macromolecules (aptamers) find growing interest as functional biopolymers for analysis, medical applications such as imaging, drug delivery and even therapeutic agents, nanotechnology, material science and more. The present perspective article introduces a glossary of examples for diverse applications of aptamers mainly originated from our laboratory. These include the introduction of aptamer-functionalized nanomaterials such as graphene oxide, Ag nanoclusters and semiconductor quantum dots as functional hybrid nanomaterials for optical sensing of target analytes. The use of aptamer-functionalized DNA tetrahedra nanostructures for multiplex analysis and aptamer-loaded metal-organic framework nanoparticles acting as sense-and-treat are introduced. Aptamer-functionalized nano and microcarriers are presented as stimuli-responsive hybrid drug carriers for controlled and targeted drug release, including aptamer-functionalized SiO_2_ nanoparticles, carbon dots, metal-organic frameworks and microcapsules. A further application of aptamers involves the conjugation of aptamers to catalytic units as a means to mimic enzyme functions “nucleoapzymes”. In addition, the formation and dissociation of aptamer-ligand complexes are applied to develop mechanical molecular devices and to switch nanostructures such as origami scaffolds. Finally, the article discusses future challenges in applying aptamers in material science, nanotechnology and catalysis.

## 1. Introduction

The eliciting of sequence-specific nucleic acids (aptamers) recognizing low-molecular-weight ligands, macromolecules and even cells by the Systematic Evolution of Ligands by Exponential Enrichment (SELEX) procedure [1,2,3,4] introduced revolutionary means to apply nucleic acids as functional materials for bioanalytical [5,6], catalytic [7,8,9] and biomedical [9,10,11,12] applications. The selective formation of aptamer-ligand complexes enabled the application of aptamers as versatile recognition elements for the development of sensor devices and sensing platforms, aptasensors [12,13,14,15]. By the immobilization of aptamers on appropriate transducers, numerous electrochemical [16,17,18,19], photoelectrochemical [20,21,22,23], surface plasmon resonance (SPR) [24,25,26], microgravimetric quartz-crystal-microbalance (QCM) [27,28,29,30], magnetic [31,32,33] and acoustic [34,35,36] sensors were demonstrated. Different target analytes were sensed by aptamers, including low-molecular-weight compounds, such as cocaine [37,38,39,40], explosives [41,42,43], pesticides [44,45], toxins [46] and antibiotics [47,48,49], and macromolecules such as protein [50,51,52,53,54,55,56,57] and biopolymers [58,59,60]. The specific recognition of metal ions by aptamers, e.g., Mg^2+^, Zn^2+^and Ni^2+^ led to the assembly of catalytic nucleic acids, DNAzymes [61,62,63], capable of cleaving oligonucleotides or ligating nucleic acids. In addition, the sequence dictated binding of transition metal complexes, such as catalytic supramolecular Fe(III)-Protoporphyrin IX (hemin) [64], or metal-ion terpyridine complexes, catalyzing chemical transformations such as tyrosine oxidation [65], and the association of chromophores, e.g., Zn (II)-Protoporphyrin IX, to sequence-specific nucleic acids, yielding photocatalysts for artificial photosynthetic transformations [66], were demonstrated. Aptamers hold great promise for biomedical applications. Beyond their use as sensing matrices, the specific recognition properties of aptamers were extensively used for targeting cell receptors, thereby facilitating the delivery of drugs and enabling cell imaging [67,68,69,70,71]. In addition, the selective binding of aptamers to cells or proteins finds growing interest for diagnostic and therapeutic applications. For example, the anti-CD30 protein aptamer was suggested for the detection of CD30 overexpressing Hodgkin lymphoma cells and as an immunotherapeutic agent for lymphoma cells [72,73,74]. Also, the anti-FX II aptamer was introduced as an antithrombosis drug by inhibiting the blood coagulation pathway [75,76]. Furthermore, the biocompatibility of aptamer nucleic acids and their low immunogenetic make the aptamers ideal drug targeting carriers, therapeutic agents and functional material for in vivo sensing applications [77,78,79].

The last two decades have introduced many different nanomaterials exhibiting unique optical, catalytic, physical adsorption and porosity properties derived from the nanodimensions and high surface area of the nanostructures. For example, the plasmonic and interplasmonic coupling characteristic to noble metal nanoparticles, such as Au or Ag nanoparticles, the catalytic and electronic properties of metal nanoparticles or carbon nanotubes and the size-controlled luminescence properties of semiconductor quantum dots, e.g., CdSe or CdTe, have been extensively used to develop many different sensing devices and imaging methods [80,81,82,83]. Not surprisingly, the integration of aptamers with nanomaterials led to the generation of aptamer-nanomaterial hybrid systems that combine the specific recognition functions of aptamers and the unique physical properties of nanomaterials. For example, the integration of aptamers with plasmonic Au nanoparticles allowed the sensing of Hg(II) [84,85,86] or thrombin [25].

In the present perspective article, we present a summary of examples originating from our laboratory demonstrating the broad applicability of aptamer-functionalized hybrid nanostructures in various scientific disciplines. Our aim is to highlight the role of aptamer-nanomaterial hybrids in the general topic of nanobiotechnology. Specific applications of aptamer-nanomaterial hybrids in developing sensors, drug-delivery carriers, new catalytic materials and supramolecular machines will be addressed. 

## 2. Hybrid Aptamer Nanostructures for Sensing Applications

The sequence-specific binding properties of aptamers have been widely applied to develop sensing platforms [12,13,14,15]. By the integration of aptamers with nanomaterials, hybrid optical sensing platforms that combine the specific recognition properties of aptamers with the unique optical or physical properties of nanomaterials were assembled [87,88,89]. This topic is introduced in this section with the integration of silver nanoclusters (AgNCs), semiconductor nanoparticles and graphene oxide. 

**Figure 1:**
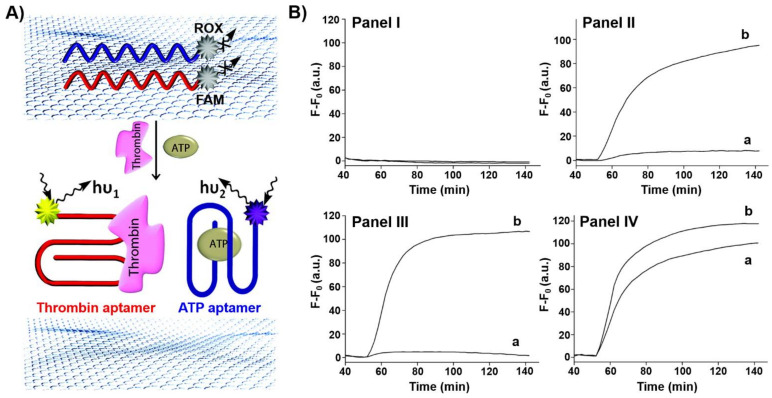
(**A**) Multiplex analysis of two ligands (thrombin, ATP) by 6-carboxyfluorescein (FAM)-functionalized thrombin aptamer and carboxy-X-rhodamine (ROX)-functionalized ATP aptamer using graphene oxide as an active support. The fluorescence of the respective fluorophores switches on after treatment with the ligands due to the desorption of the respective aptamer-ligand complexes from the graphene oxide. (**B**) Time-dependent fluorescent changes in the solution: Panel I—in the absence of ligands, Panel II—in the presence of thrombin, Panel III—in the presence of ATP, IV—in the presence of ATP and thrombin. a corresponds to the fluorescence of ROX and b of FAM. Reproduced with permission from ref. [90]. Copyright 2012 American Chemical Society. Arrows: the arrows correspond to typical excitation and emission arrows.

Single stranded nucleic acids adsorb strongly to graphene oxide nanosheets. Accordingly, aptamers were functionalized with fluorophores and adsorbed onto graphene oxide nanosheets. The fluorophore labels were quenched by the graphene oxide, yet desorption of the aptamers from the graphene oxide carrier, through the formation of aptamer-ligand complexes, switched on the fluorescence of the labels, thus allowing the optical detection of the ligands. This is exemplified in Figure 1A with the multiplexed analysis of thrombin and ATP by the respective aptamers [90]. The ATP aptamer was labeled with the carboxy-X-rhodamine (ROX) fluorophore (λ_em_ = 603 nm), whereas the thrombin aptamer was labeled with the 6-carboxyfluorescein (FAM) fluorophore (λ_em_ = 518 nm). The labeled aptamers were adsorbed onto graphene oxide and their fluorescence was quenched. The multiplexed analysis of the ATP and thrombin analytes is presented in Figure 1B. While in the absence of the analyte no fluorescence in the solution was detected (Panel I). In the presence of thrombin the fluorescence of FAM was switched on in the solution (Panel II), and in the presence of ATP the fluorescence of ROX was switched on (Panel III). In the presence of ATP and thrombin, both aptamer-ligand complexes were desorbed into the solution resulting in the fluorescence of FAM and ROX.

**Figure 2:**
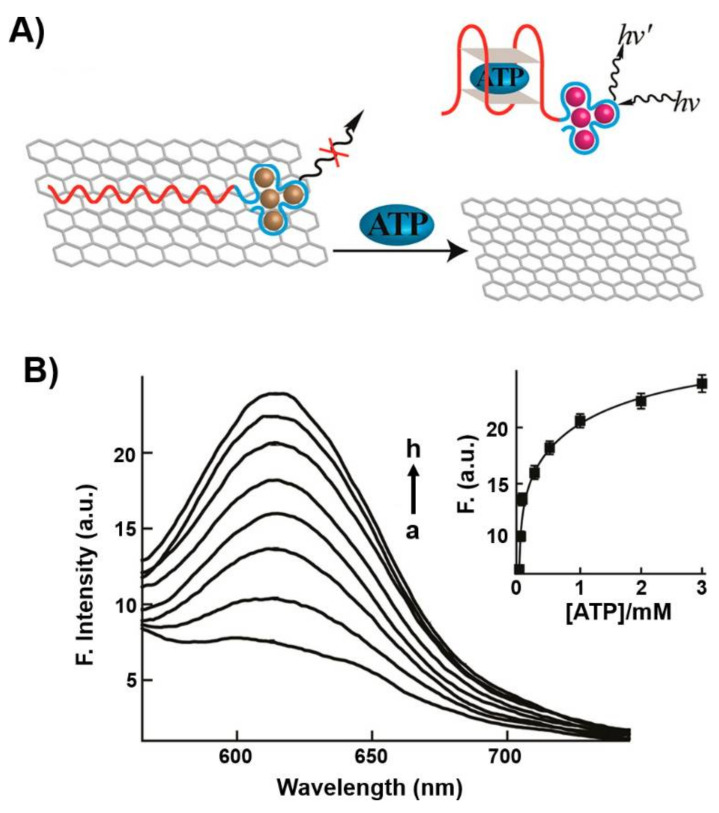
(**A**) Application of graphene oxide modified with ATP aptamer conjugated to nucleic acid-stabilized silver nanoclusters (AgNCs) as a hybrid system for the analysis of ATP. The fluorescence of the AgNCs is quenched in the aptamer/graphene oxide hybrid structure and it is switched on upon the desorption of the AgNCs-ATP aptamer/ATP complex from the graphene oxide support. (**B**) Fluorescence spectra generated upon desorption of the AgNCs-ATP aptamer/ATP complex in the presence of different concentrations of ATP for a fixed time-interval of 1 h. a = 0 mM; b = 0.025 mM; c = 0.05 mM; d = 0.25 mM; e = 0.5 mM; f = 1 mM; g = 2 mM; h = 3 mM. Inset: derived calibration curve. Reproduced with permission from ref. [91]. Copyright 2013 American Chemical Society. Arrows: the arrows correspond to typical excitation and emission arrows.

In a related system, the fluorescent properties of sequence-specific nucleic acid-stabilized silver nanoclusters (AgNCs) provided means to design optical aptosensors [91]. Nucleic acid-stabilized AgNCs, λ_em_ = 616 nm, were extended by the ATP aptamer sequence to yield a functional probe for sensing ATP. The probe was adsorbed onto graphene oxide resulting in the quenching of the fluorescence of the AgNCs. In the presence of ATP, the probe was desorbed from the graphene oxide support leading to the switching on of fluorescence of the NCs (λ = 616 nm) (Figure 2A). The fluorescent spectra of the desorbed AgNCs-ATP aptamer/ATP complex at different concentrations of ATP, and the resulting calibration curve, are displayed in Figure 2B. The sensing of ATP by the hybrid nucleic acid/AgNCs/graphene oxide was specific and the sensing matrix did not respond to other nucleotide triphosphates. The sensing platform was, similarly, applied to sense thrombin using a different-sized nucleic acid-stabilized fluorescent AgNCs (λ = 775 nm) conjugated to the thrombin aptamer and adsorbed onto graphene oxide.

**Figure 3:**
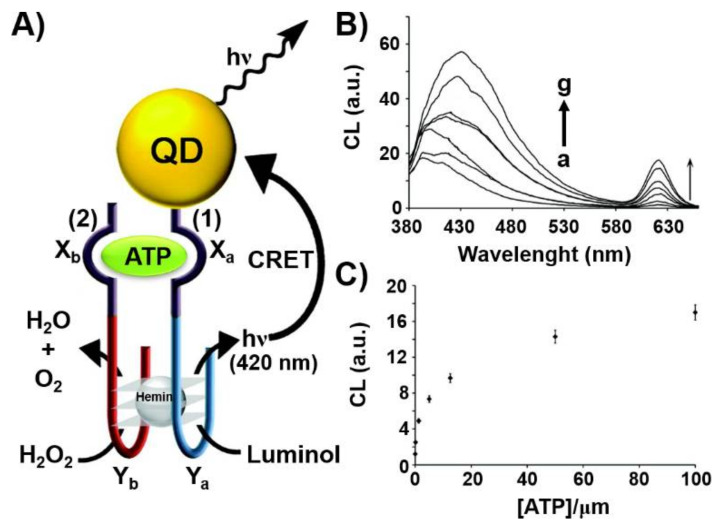
(**A**) Chemiluminescence resonance energy transfer (CRET)-induced sensing of ATP through the ATP-induced assembly of a supramolecular hemin/G-quadruplex-ATP aptamer/ATP complex on a semiconductor CdSe/ZnS quantum dot (QD). The hemin/G-quadruplex catalyzed oxidation of luminol in the presence of H_2_O_2_ yields chemiluminescence that results in the CRET process to the QDs and in the luminescence of the QDs. (**B**) CRET-induced luminescence spectra of the QDs, λem = 620 nm, in the presence of different concentrations of ATP: a = 0 μM; b = 0.125 μM; c = 1.25 μM; d = 5 μM; e = 12.5 μM; f = 50 μM; g = 100 μM. (**C**) Derived calibration curve for the CRET-induced analysis of ATP by the nucleic acid/QD hybrid. Reproduced with permission from ref. [92]. Copyright 2011 American Chemical Society. Arrows: the arrows correspond to typical excitation arrows.

Another optical nanostructure-based aptasensor is displayed in Figure 3 [92]. Figure 3A depicts the chemiluminescence resonance energy transfer (CRET)-stimulated sensing of ATP using a supramolecular hemin/G-quadruplex-ATP aptamer-CdSe semiconductor quantum dot (QD) hybrid module. The sensing platform made use of the chemiluminescence generated by the hemin/G-quadruplex catalyzed oxidation of luminol by H_2_O_2_ and the subsequent CRET process to the CdSe QDs that leads to the luminescence of the QDs without external irradiation. Accordingly, CdSe QDs were modified with the nucleic acid sequence (**1**) that included the ATP aptamer subunit, X_a_, and the G-quadruplex subunit, Y_a_. In the presence of the coadded strand (**2**), composed by the complementary ATP aptamer sequence, X_b_, and the G-quadruplex subunit, Y_b_, and ATP analyte, K^+^-ions and hemin, the cooperative stabilization of the supramolecular ATP/ATP aptamer complex-hemin/G-quadruplex on the QDs carrier occurred. The hemin/G-quadruplex-catalyzed oxidation of luminol by H_2_O_2_ generated chemiluminescence stimulating the CRET process to the QDs and the consequent luminescence of the QDs at λ = 620 nm. As the efficiency of the CRET process was controlled by the concentration of ATP forming the supramolecular catalytic nanostructure on the QDs, the resulting CRET signal allowed the quantitative analysis of ATP, Figure 3B,C.

**Figure 4:**
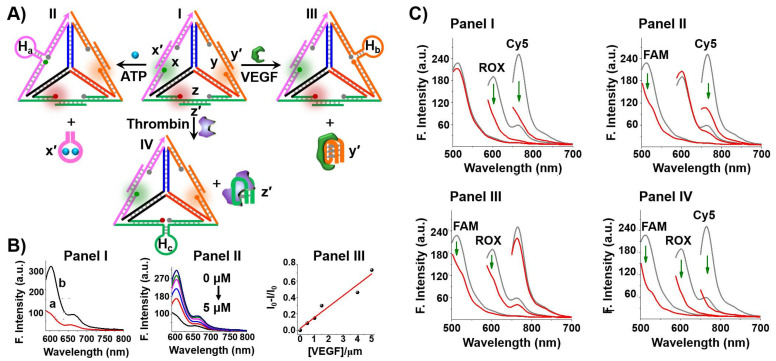
(**A**) Multiplex analysis of three different ligands (ATP, VEGF and thrombin) using a functional DNA tetrahedron module (I) functionalized at the three edges with ATP aptamer (x’), VEGF aptamer (y’) and thrombin aptamer (z’). The aptamer units stretch the fluorophore-quencher functionalized edges (x, y, z) into fluorescent configurations (x-FAM, y-ROX, z-Cy5). The formation of the respective aptamer-ligand complexes led to the displacement of the x’-ATP, y’-VEGF and z’-thrombin complexes and to the distortion of the respective edges into fluorophore-quenched configuration allowing the multiplex analysis of the respective ligands and their quantitative sensing. (**B**) Example for the fluorescence analysis of VEGF through the distortion of edge y’ and quenching of ROX. Panel I—fluorescence of ROX in the DNA tetrahedron module: a. before addition of VEGF; b. after addition of VEGF, 5 μM. Panel II—fluorescence of ROX in the DNA tetrahedron module upon sensing of different concentrations of VEGF for a fixed interval of time. Panel III—derived calibration curve for sensing VEGF with the DNA tetrahedra module. (**C**) Multiplex analysis of the different ligands by the DNA tetrahedron module. Fluorescence changes upon sensing: Panel I—VEGF and thrombin; Panel II—ATP and VEGF; Panel III—ATP and thrombin; Panel IV—ATP, VEGF, and thrombin. Arrows represent the decrease in the fluorescence. Reproduced with permission from ref. [102]. Copyright 2020 American Chemical Society.

A different supramolecular nanostructure for the optical detection of aptamer-ligand complexes included DNA tetrahedra nanostructures. DNA three dimensional (3D) tetrahedra nanostructures have attracted substantial recent interest in DNA nanotechnology. The ease to self-assemble different-sized DNA tetrahedra structures with high yields from pre-engineered single strands exhibiting appropriate complementarities provides versatile 3D structures for different applications [93,94,95]. The integration of DNAzymes into the edges of the tetrahedra, the tethering of nucleic acids to the corners of the tetrahedra nanostructures [96] and the demonstration of the superior cell permeation [97,98,99] provide versatile methods for cell targeting and recognition events. Indeed, the DNA tetrahedra nanostructures were extensively used for biomedical applications, including the development of sensors and cell imaging [100,101]. Here, we present the application of an aptamer-functionalized supramolecular DNA tetrahedron nanostructure as a functional module for the multiplexed analysis of aptamer-ligand complexes (Figure 4A) [102]. A DNA-tetrahedron module, I, consisting of edges x, y, z stabilized by three spatially-separated fluorophore-quencher pairs (fluorophores = FAM/quencher BHQ1 edge x; ROX/quencher BHQ2 edge y and Cy5/quencher BHQ2 edge z) was prepared. The edges were stretched by hybridization with the respective aptamer, ATP aptamer (x’) VEGF (vascular endothelial growth factor) aptamer (y’) and thrombin aptamer (z’). In the spatially-aptamer stretched tetrahedron module the fluorescence of the respective fluorophores was switched on. Subjecting the sensing module to any of the aptamer ligands (ATP, VEGF, thrombin) resulted in the displacement of the respective aptamer-ligand complexes and in the reconfiguration of the edges into hairpin structures, H_a_, H_b_ or H_c_, where the fluorophore-quencher pairs are forced into an intimate position leading to the quenching of the respective fluorophore. Figure 4B exemplifies the sensing of variable concentrations of VEGF by the sensing module. Similar selective sensing of ATP and thrombin were demonstrated by the reconfiguration of the edges x or z in the presence of ATP or thrombin, respectively. In addition, the DNA tetrahedron sensing module was successfully applied for the multiplex analysis of the different analytes, Figure 4C.

**Figure 5:**
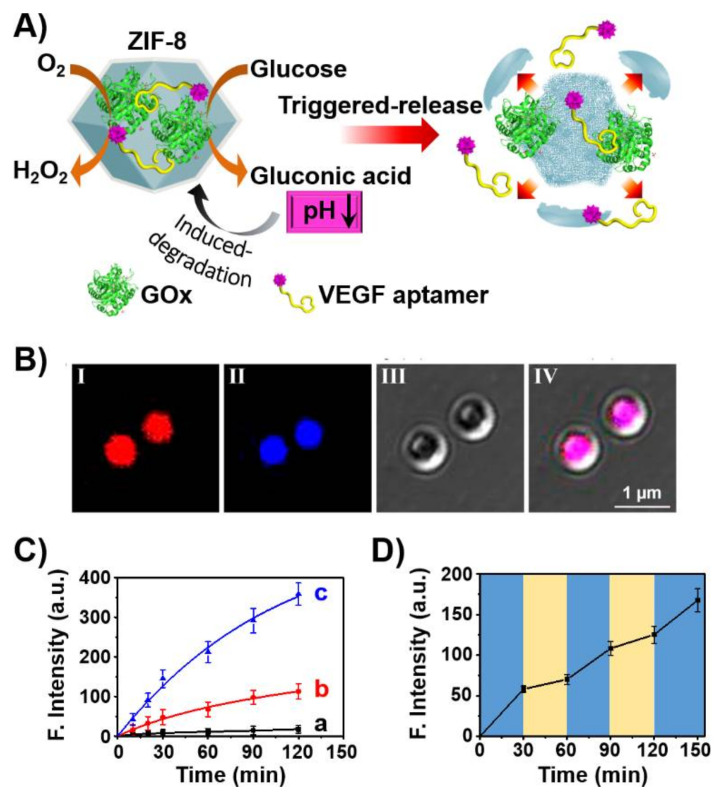
(**A**) Schematic glucose-stimulated release of VEGF aptamer from glucose oxidase (GOx)-loaded ZIF-8 metal-organic framework nanoparticles (NMOFs). (**B**) Confocal microscopy images of the Cy3-labeled VEGF aptamer (I) and coumarin-labeled GOx (II) and bright field (III) and merged images (IV) of the loaded NMOFs. (**C**) Time-dependent release of the Cy3-labeled VEGF aptamer from the GOx/Cy3-labeld VEGF aptamer-loaded ZIF-8 NMOFs at different concentration of glucose: a = 0 mM, b = 10 mM, c = 50 mM. (**D**) Switchable release of Cy3-labeled VEGF aptamer from the GOx/VEGF aptamer-loaded ZIF-8 NMOFs in the presence of high concentration of glucose (15 mM) and low concentration of glucose (5 mM). Reproduced with permission from ref. [103]. Copyright 2018 American Chemical Society. Arrows: it indicates a decrease in the pH.

The use of aptamers as therapeutic materials was further addressed by the incorporation of aptamers in stimuli-responsive nanocarriers that allow the triggered release of the aptamer by appropriate cellular conditions. This is exemplified in Figure 5 with the assembly of ZIF-8 metal-organic framework nanoparticles (NMOFs), loaded with glucose oxidase (GOx) and the VEGF aptamer [103]. The ZIF-8 NMOFs, composed of Zn^2+^-ions interbridged by 2-methylimidazole, are pH-sensitive, and at pH < 5.5 are degraded. Thus, subjecting the GOx/VEGF-loaded NMOFs to glucose results in the aerobic GOx-catalyzed oxidation of glucose to gluconic acid that acidifies the metal-organic framework. The acidic pH leads to the degradation of the NMOFs and the release of the VEGF aptamer that acts as an inhibitor of angiogenesis promoted by VEGF. The integration of the GOx/VEGF aptamer in the NMOFs was confirmed by labeling of the VEGF aptamer with Cy3 and GOx with coumarin and imaging the NMOFs by confocal fluorescence microscopy (Figure 5B). The glucose-stimulated release of the VEGF aptamer was controlled by the concentration of glucose and, as the concentration of glucose increased, the release of the VEGF aptamer was enhanced (Figure 5C). This suggests the potential application of the GOx/VEGF-loaded NMOFs for ocular therapeutic treatment via the dose-controlled inhibition of the VEGF-induced angiogenesis blood vessel clotting in the eyes. That is, at high intraocular glucose concentration, the degradation of the NMOFs is enhanced leading to the effective release of the VEGF aptamer and inhibition of VEGF. Nonetheless, at a lower concentration of glucose, the local pH changes in the NMOFs are readily dissipated by the buffered solution of the eye containment leading to the blockage of ZIF-8 decomposition and to the release of the aptamer. Thus, the intraocular concentration changes could be envisaged as triggers for the dose-controlled release of the VEGF aptamer in the eye, Figure 5D.

## 3. Aptamers as Responsive Gates for Nano or Microcarriers

Different types of carriers are used for drug delivery and controlled release. These include nanoparticles, such as porous silica nanoparticles [104,105,106], porous metal organic framework particles [107,108] and Au nanoparticles [109], and carbon-based materials such as graphene oxide nanoparticles [110] and C-dots [111]. In addition, carriers such as liposomes [112,113], microcapsules [114,115,116,117] or polymer particles [118,119,120] have been used as drug delivery systems. Specifically, the chemical modification of these carriers with stimuli-responsive gating units provides versatile means to stimulate the target controlled and, eventually, switchable drug release. Different gating units being uncaged by different chemical triggers such as pH [103,121,122], redox-units [123,124] or physical stimuli such as heat [125,126,127], light [128,129,130], magnetic fields [131,132], ultrasound [133,134,135] or microwaves [136], were reported. 

Nucleic acid structures have been often used as caging units of drug carriers that can be unlocked by nucleic acid biomarkers such as miRNAs [137], the pH-induced separation of DNA locks via the dissociation of i-motifs [138,139] or triplexes, [140,141] the separation of K^+^-ion stabilized G-quadruplex locks by crown ether [142,143], and the degradation of DNA locks by enzymes [144] or DNAzymes [145]. Physical triggers, such as heat [146,147,148] or light [149,150,151,152,153] have also been used to uncage DNA-gated carriers. The sequence-specific recognition properties of aptamer-ligand complex have been extensively used to design nucleic acid-based gating units being unlocked through the formation of aptamer-ligand complexes [154]. These unlocking principles have specific merit since many ligands act as biomarkers for diseases, and then the biomarker-induced uncaging of the carriers may act as autonomous sense-and-treat systems for the target-controlled release of drugs. Here, we provide several examples of aptamer-gated carriers for the controlled release of drugs.

**Figure 6:**
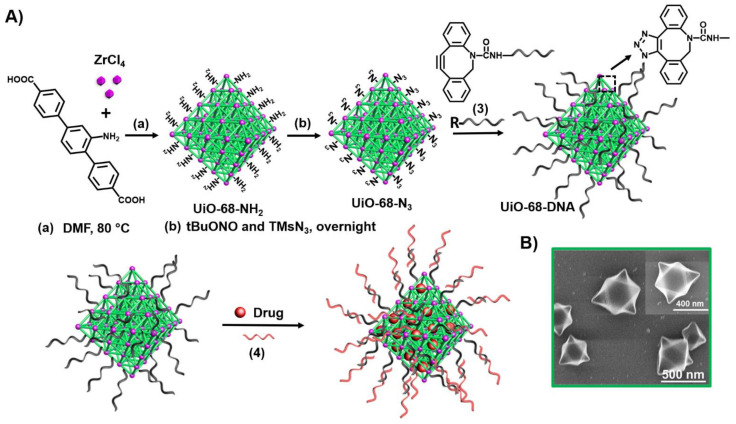
(**A**) Synthesis of nucleic acid-functionalized UiO-68 NMOFs loaded with a dye or a drug and locked by means of a stimuli-responsive strand (**4**). (**B**) SEM images of the nucleic acid-modified NMOFs.

Metal-organic framework nanoparticles (NMOFs) provide a broad class of porous materials with high loading capacity of drugs [107]. Figure 6 exemplifies the stepwise synthesis of the NMOFs and their gating with ATP aptamer locks [155]. Azide-modified UiO-68 NMOFs were functionalized with DBCO-modified nucleic acid (**3**) (DBCO = dibenzocyclooctyne) using the click chemistry principle. The NMOFs were loaded with the anticancer drug doxorubicin (DOX) and the loaded NMOFs were locked by hybridization with the ATP aptamer (**4**). 

**Figure 7:**
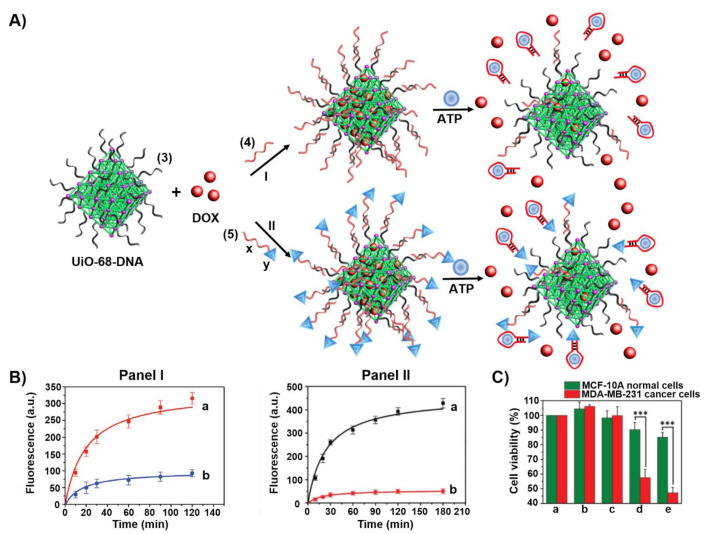
(**A**) Synthesis of doxorubicin (DOX)-loaded ATP aptamer-gated UiO-68 NMOFs and the ATP-driven release of the drug from the NMOFs through the formation of ATP/ATP aptamer complexes; (I) locking of the NMOFs with the ATP aptamer (**4**) (II) locking of the NMOFs by the ATP aptamer conjugated to the AS1411 aptamer as cancer cell targeting unit (**5**). (**B**) Time-dependent release of DOX from: Panel I—ATP aptamer-gated NMOFs: a. in the presence of ATP, 25 nM; b. in the absence of ATP. Panel II—ATP/AS1411 aptamer-gated NMOFs: a. in the presence of ATP, 25 mM; b. in the absence of ATP. (**C**) Cytotoxicity of the DOX-loaded ATP-responsive NMOFs towards normal MCF-10A epithelial breast cells (green) and MDA-MB-231 breast cancer cells (red) and appropriate control experiments: a. = untreated cells; b.= unloaded ATP aptamer-modified NMOFs; c = unloaded ATP/AS1411 aptamer-modified NMOFs; d = ATP aptamer-modified NMOFs loaded with DOX; e = ATP/AS1411 aptamer-modified NMOFs loaded with DOX (cells treated for a time-interval of three days). Data are averages ± SD (*n* = 3 experimental replicates). *** *p* < 0.001 by t-test versus normal cells. Reproduced with permission from ref. [155]. Copyright 2017 Wiley-VCH.

Figure 7 outlines two configurations of the caged ATP-responsive NMOFs and presents schematically the release of the drugs [155]. In one configuration, path I, subjecting the NMOFs to the ATP ligand results in the formation of the ATP/ATP aptamer complexes and the release of the drug. In the second configuration, path II, the ATP aptamer (x) is further elongated with the AS1411 aptamer sequence (y), that binds to the nucleolin receptor associated with different cancer cells as a targeting element, sequence (**5**). Figure 7B shows the time-dependent release of DOX from the (**3**)/(**4**)-gated NMOFs, panel I, and from the (**3**)/(**5**)-gated NMOFs, panel II. The cytotoxicity of the different NMOFs towards MDA-MB-231 breast cancer cells as compared to normal MCF-10A epithelial breast cells is addressed in Figure 7C. While the normal epithelial cells are almost unaffected by the NMOFs, effective cell death of the MDA-MA-231 cancer cells by the (**3**)/(**4**)-gated NMOFs (45% cell death) and by the (**3**)/(**5**)-gated NMOFs (≈60% cell death) was observed. The cytotoxicity of the gated NMOFs towards the cancer cells was attributed to the over-expressed ATP in the cancer cells that resulted in the effective unlocking and release of the drug from the carriers. The enhanced cytotoxicity of the (**3**)/(**5**)-gated NMOFs was attributed to the AS1411 targeting of the carriers to the cells and to the facilitated permeation of the carriers into the cancer cells.

Other aptamer-gated NMOFs were developed following the protocol previously described including VEGF aptamer-functionalized DOX-loaded NMOFs for the release of a drug by over-expressed VEGF in cancer cells, [156] and thrombin aptamer-functionalized Apixaban-loaded NMOFs for the thrombin triggered release of the antiblood-clotting drug through the formation of the thrombin/thrombin aptamer complexes [157].

**Figure 8:**
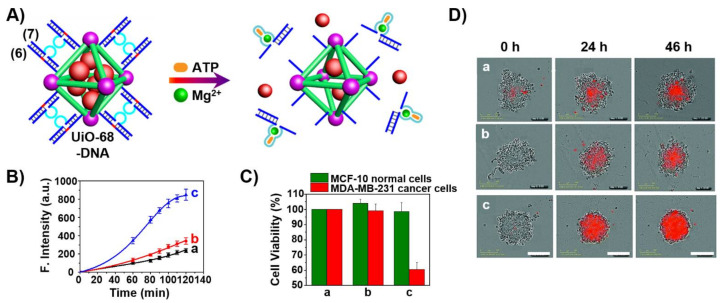
(**A**) DOX-loaded UiO-68 NMOFs gated by the strands (**6**)/(**7**), and their cooperative unlocking in the presence of Mg^2+^ and ATP. Strand (6) contains the ribonucleobase corresponding to substrate of the Mg^2+^-dependent DNAzyme and strand (**7**) includes the Mg^2+^ dependent DNAzyme sequence integrated with the ATP aptamer sequence. (**B**) Time-dependent release of DOX from the (**6**)/(**7**)-locked DOX-loaded UiO-68 NMOFs: (a) in the absence of the Mg^2+^-ions and ATP. (b) only in the presence of Mg^2+^-ions, 2 mM. (c) in the presence of Mg^2+^-ions, 2 mM and ATP, 3 mM. (**C**) Cytotoxicity of the (**6**)/(**7**)-locked DOX-loaded NMOFs towards MCF-10A normal epithelial breast cells (green) and MDA-MB-231 breast cancer cells (red): a = untreated cells, b = (**6**)/(**7**)-locked, unloaded NMOFs, c = (**6**)/(**7**)-locked, DOX-loaded NMOFs. (**D**) Typical apoptosis color images after 0, 24 and 46 h corresponding to = a. untreated cell aggregates; b = cell aggregates treated with (**6**)/(**7**)-locked, unloaded NMOFs; c = cell aggregates treated with (**6**)/(**7**)-locked, DOX-loaded NMOFs. Reproduced with permission from ref. [145]. Copyright 2017 Royal Society of Chemistry.

In a further approach [145], the UiO-68 NMOFs loaded with DOX were locked with duplex nucleic acids (**6**)/(**7**), where the strand (**7**) includes a loop domain composed of the ATP aptamer sequence (light blue) separated by two subunits (dark blue) that correspond to the Mg^2+^-ion-dependent DNAzyme sequence, and the strand (**6**) includes the ribonucleobase containing sequence that corresponds to the substrate of the Mg^2+^-ion-dependent DNAzyme. The flexibility of the loop region of (**7**) prohibits the formation of the active Mg^2+^-ion-dependent DNAzyme. In the presence of ATP, the loop domain reconfigures into the ATP/ATP aptamer ligand complex that results in the rigidification of loop region and the active Mg^2+^-dependent DNAzyme structure. This leads to the ATP-triggered activation of the DNAzyme that catalyzes the cleavage of the substrate, the sequestered dissociation of the locks and the release of the loaded drug (Figure 8A). Figure 8B reveals that the carrier is unlocked only in the presence of ATP and Mg^2+^-ions that act as cooperative triggers for the release of the drug. Cytotoxicity experiments revealed that the over-expressed ATP in MDA-MB-231 breast cancer cells stimulated ca. 45% cell death after treatment of the cells with the DOX-loaded (**6**)/(**7**)-locked NMOFs while normal epithelial breast cells were unaffected under similar conditions (Figure 8C). Similar cytotoxic effects were observed upon following colorimetrically the time-dependent apoptosis of spheroid MDA-MB-231 aggregates and appropriate control systems (Figure 8D). The cytotoxicity and downstream in vivo degradation of NMOFs and consequent release of metal ions and bridging ligands were addressed in several studies [158,159]. Although these effects should be examined for each specific NMOF carrier, the available reports claimed minute toxicity of the particles or degraded metal ions/ligands (e.g., ZIF-8 NMOFs).

**Figure 9:**
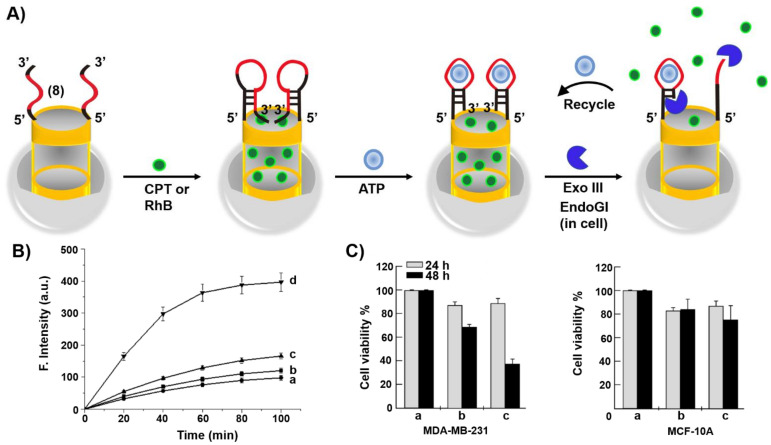
(**A**) Hairpin (**8**)-gated, camptothecin (CPT) loaded MP SiO_2_ NPs for the ATP-driven and exonuclease III (Exo III)-amplified release of loads. The hairpin (**8**) contains the ATP aptamer sequence in a caged structure. In the presence of ATP, the hairpin (8) is reconfigured into an ATP/ATP aptamer supramolecular structure that is cleaved by Exo III, leading to the degradation of the locks and the recycling of ATP for the further unlocking of the gate units (amplification step). (**B**) Time-dependent release of CPT from the (**8**)-hairpin-caped NPs: a = in the absence of ATP and Exo III; b = in the presence of ATP (1 mM); c = in the presence of Exo III (0.5 U/μL); d = in the presence of ATP (1 mM) and Exo III (0.5 U/μL). (**C**) Cytotoxicity of the CPT-loaded MP SiO_2_ NPs towards MDA-MB-231 breast cancer cells in comparison to normal epithelial MCF-10A breast cells. a = nontreated cells; b = cells treated with free CPT; c= cells treated with the CPT-loaded MP SiO_2_ NPs. Reproduced with permission from ref. [161]. Copyright 2013 American Chemical Society.

Mesoporous SiO_2_ nanoparticles (MP SiO_2_ NPs) provide a versatile carrier for drug delivery [160]. This is exemplified in Figure 9A with the loading of the nucleic acid (**8**)-functionalized MP SiO_2_ NPs with the anticancer drug camptothecin, CPT [161]. The heated (**8**)-functionalized NPs (to retain the strand in an open flexible configuration) were loaded with CPT and the cooled-down of the carriers resulted in the reconfiguration of (**8**) into a hairpin structure that locked the drug in the pores of the NPs. The hairpin loop structure and part of the stem domain were engineered to include the ATP aptamer sequence. In the presence of ATP, the hairpin structure was reconfigured to yield the ATP/ATP aptamer complex in a hairpin structure that includes the 3′-end of the aptamer in a fully hybridized structure with the counter stem domain. In the presence of Exonuclease III, Exo III, the hairpin stem domain is hydrolytically digested, resulting in the uncaging of the hairpin locks, leading to the release of ATP and CPT. The Exo III cleavage of the hairpin structure provides a path to regenerate ATP for further unlocking events. Figure 9B, curve (d), depicts the time-dependent release of CPT from the drug-load carrier in the presence of Exo III. Control experiments demonstrated inefficient release of the drug in the absence of Exo III or upon exclusion of the ATP. Cytotoxicity experiments (Figure 9C) indicated that ca. 60% cell death of MDA-MB231 breast cancer cells treated with the CPT loaded NPs was observed after a time-interval of 48 h, while treatment of MCF-10A normal epithelial cells with the CPT-loaded carrier NPs showed only 20% cell death under similar conditions. The effective cytotoxicity of the CPT-loaded NPs towards the cancer cells was attributed to the over-expressed levels of ATP in the cancer cells that stimulated the effective release of the drug from the carrier NPs.

**Figure 10:**
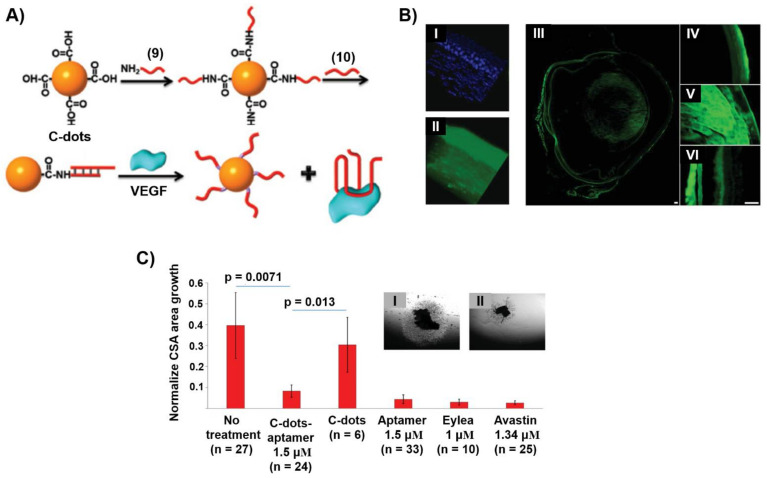
(**A**) VEGF aptamer-modified C-dots as functional carriers for the VEGF-induced release of the aptamer and the formation of VEGF/VEGF aptamer complexes that inhibit the angiogenetic function of VEGF. (**B**) Penetration of C-dots into the various eye structures and noninvasive monitoring of the intraocular concentration of C-dots following topical administration. C-dots topically applied on the cornea penetrated all eye layers and structures. Confocal microscopy of a cornea topically treated with C-dots exhibiting characteristic C-dots high fluorescence in the corneal epithelium and stroma (I, II). A gross histology of the eye showing a wide distribution of the C-dots along the entire eye structures (III), including the cornea (IV), the lens (V) and vitreous, retina and choroid (VI) (**C**) Results probing the angiogenetic effect of the VEGF aptamer-modified C-dots in comparison to commercial therapeutic drugs. Inset. Panel I-growth and sprouting of the blood vessel in choroids treated with C-dots lacking the VEGF aptamer. Panel II-inhibition of blood vessel sprouting in choroids treated with the VEGF aptamer-modified C-dots. Reproduced with permission from ref. [170]. Copyright 2019 Wiley-VCH.

Carbon nanomaterials, such as carbon nanotubes, graphene oxide or porous carbon nanoparticles have attracted substantial interest as functional materials for different applications [162,163,164]. Within this class of nanomaterials, carbon nanoparticles, C-dots, are of particular interest due to their high surface area, surface functionalities, allowing the binding of ions or ligands, and photophysical luminescence functions [165]. These properties allowed the use of C-dots as catalysts [166] or sensors [167,168]. In addition, the modification of C-dots, their low cytotoxicity and effective cell permeation properties allowed their use as drug carriers [169]. As aptamers are promising modules for therapeutic applications, the use of C-dots as a support for carrying aptamers seems an attractive path to apply aptamer/C-dots hybrids for drug delivery and therapeutic applications. This is exemplified with the application of C-dots modified with the VEGF aptamer for topical treatment of ocular disorders [170]. VEGF induces angiogenetic driven diseases associated with age-related macular degeneration (AMD) and diabetic retinopathy (DR), causing blindness. The inhibition of the VEGF angiogenetic process through binding of VEGF to the VEGF aptamer is a recently viable therapy yet limited by the need of repeated intraocular injections. Figure 10A depicts the modification of carboxylic acid-functionalized C-dots with the VEGF aptamer. The C-dots were covalently modified with the amine-functionalized nucleic acid (**9**) to which the VEGF aptamer (**10**) was hybridized. This hybrid carrier was designed as a functional platform for carrying the aptamer across the corona, into the retina and posterior sclera, where the VEGF-induced release of the aptamer is anticipated to form the G-quadruplex VEGF aptamer-VEGF complex, leading to the inhibition of the protein. Optical measurements that followed the fluorescence of the C-dots demonstrated the effective permeation of the (**9**)/(**10**)-functionalized C-dots across the various eye structures and their accumulation in the retina and choroid (Figure 10B). In addition, no cytotoxic effects of the C-dots were observed and the inhibition function of the aptamer/C-dots hybrid was demonstrated using an in vitro model of choroidal vascular angiogenesis (Figure 10C). The VEGF aptamer-loaded C-dots revealed an obvious inhibitory effect on choroidal blood vessel growth and sprouting as compared to nontreated samples, and their effect was comparable to commercial therapeutic drugs administered, at present, by invasive injection. That is, the aptamer/C-dots hybrid has the potential of topical administration to resolve a major problem in diseases.

**Figure 11:**
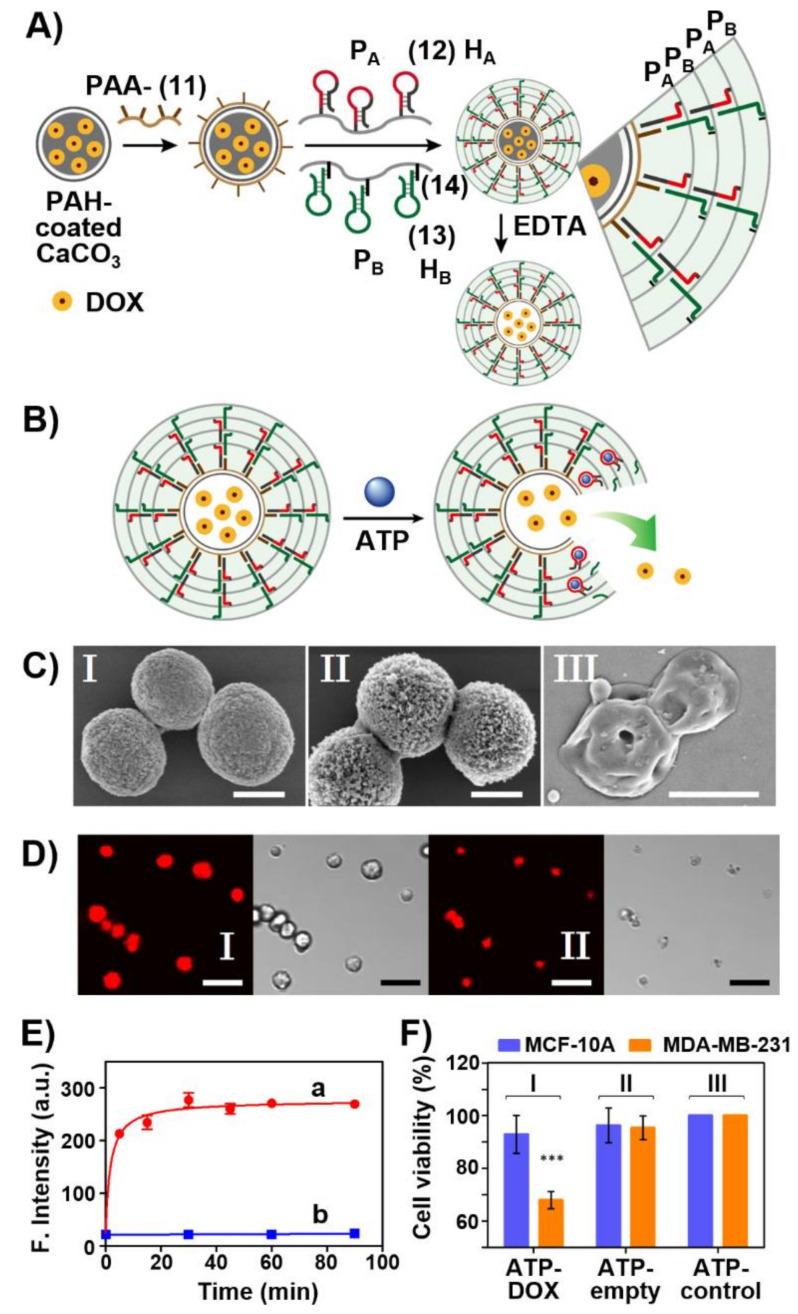
(**A**) Schematic preparation of doxorubicin-dextran (DOX-D)-loaded microcapsules stabilized by an ATP-responsive hydrogel shell created using the hybridization chain reaction. (**B**) ATP-driven release of DOX-dextran by degradation of the hydrogel shell. (**C**) SEM images of: I—uncoated DOX-D-loaded CaCO_3_ microparticles; II—DOX-D-loaded ATP aptamer-bridged hydrogel-coated CaCO_3_ microparticles; III—DOX-D-loaded ATP-responsive hydrogel-coated microcapsules after the dissolution of the CaCO_3_ core (panel III). Scale bars, 2 μm (I and II) and 1 μm (III). (**D**) Confocal fluorescence microscopy images and bright-field microscopy images of DOX-D-loaded ATP aptamer-bridged hydrogel microparticles before (I) and after (II) the dissolution of the core. Scale bar is 10 μm. (**E**) Time-dependent release of DOX-D from the microcapsules: a. in the presence of ATP (50 mM); b. in the absence of ATP. (**F**) Cytotoxicity the DOX-D-loaded hydrogel-coated microcapsules towards MDA-MB-231 breast cancer cells (orange) and normal epithelial breast cells (blue) incubated with the microcapsules for 6 h (I), and corresponding control experiments (treatment of the cells with unloaded microcapsules (II) and nontreated cells. (III)). *** denotes *p* < 0.001 Reproduced with permission from ref. [171]. Copyright 2017 Royal Society of Chemistry.

A further versatile aptamer-responsive, therapeutically-promising drug carrier system includes aptamer-gated hydrogel microcapsules [171]. Figure 11A depicts the construction of drug-loaded microcapsules and the schematic unlocking of the microcapsules through the formation of aptamer-ligand complexes, where the ligand is a biomarker for a disease. Calcium carbonate microparticles were impregnated with DOX-dextran (DOX-D) as an anticancer drug. The DOX-D-loaded CaCO_3_ microparticles were coated with positively charged polyallylamine hydrochloride (PAH). Polyacrylic acid (PAA) modified with the amino nucleic acid (**11**) was coadsorbed by electrostatic interactions onto the positively charged PAH-coated particles. The polyacrylamide polymer chains P_A_ and P_B_ were functionalized with hairpin H_A_, (**12**), and H_B_ (**13**) linked to the polymer through a short tether (**14**) (for directionality reasons). The hairpin H_A_ includes in its stem region and the loop domain the ATP aptamer sequence and, in addition, a part of the loop domain includes the complementary sequence for the promoter (**11**) associated with the polymer coated microparticles. The hairpins H_A_ and H_B_ are engineered to allow inter communication so that the open hairpin H_A_ includes a toehold tether to open hairpin H_B,_ and vice versa, the opened hairpin H_B_ includes a toehold tether to open hairpin H_A_. Thus, subjecting the (**11**)-modified microparticles to the polymers P_A_ and P_B_ led to the (**11**)-induced hybridization chain reaction (HCR) where (**11**) opens hairpin H_A_ associated with P_A_ and the resulting open H_A_ units open the hairpin H_B_ associated with P_B_ and vice versa, resulting in a polyacrylamide hydrogel coating crosslinked by the duplex formed between (**12**) and (**13**). Subsequently, the CaCO_3_ core microparticles were dissolved in the presence of EDTA to yield DOX-D-loaded microcapsules. As the duplex (**12**)/(**13**) bridging units include the ATP aptamer sequence, in the presence of ATP, the duplex units are separated by forming the (**12**)-ATP aptamer/ATP (ligand) complexes, resulting in the perforation of the hydrogel coating and the release of the DOX-D-loaded drug (Figure 11B). The resulting DOX-D-loaded microcapsules before and after etching of the CaCO_3_ cores were imaged by scanning electron microscopy (SEM) (Figure 11C) and confocal fluorescence and bright-field microscopy (Figure 11D). Figure 11E shows the ATP-stimulated unlocking of the microcapsules, while in the absence of ATP the release of DOX-D is effectively blocked, uncaging of the microcapsules and release of DOX-D is observed in the presence of ATP. The release of ATP is controlled by the concentration of ATP, and as the concentration of ATP increased the release process was enhanced. Furthermore, control experiments reveal specificity toward ATP as uncaging agent, and other nucleotide triphosphate (CTP, GTP, TTP) did not affect the release process. As ATP is over-expressed in cancer cells, it could act as trigger to release the drug in cancer cells. Indeed, cytotoxicity experiments evaluating the effect of the DOX-D-loaded microcapsules on MDA-MB-231 breast cancer cells and control MCF-10A normal epithelial breast cells revealed obvious cytotoxic efficacy of the microcapsules towards the cancer cells (Figure 11F). Treatment of the MDA-MB-231 cancer cells and the MCF-10A normal cells with the DOX-D-loaded microcapsules resulted in 35% cell death of the cancer cells and only 10% cell death of the normal cells after five days of treatment. The superior cytotoxicity of the microcapsules towards the cancer cells was attributed to the elevated contents of ATP in the cancer cells and to the improved permeation of the microcapsules into the cancer cells as compared to the normal cells. Although the origin for the superior permeation of the microcapsules into the cancer cells is, at present, not fully understood, we believe that the enhanced porosity of the cancer cell membrane facilitates the enhanced permeability and retention effect (EPR) transport through the cancer cell boundaries.

## 4. Aptamer Nanostructures for Catalysis

**Figure 12:**
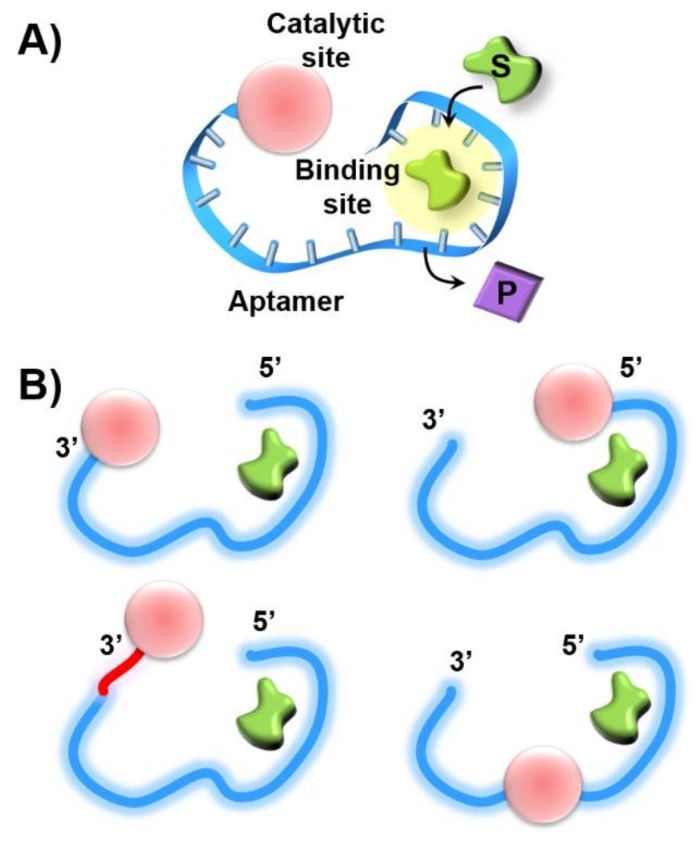
(**A**) Schematic structure of a nucleoapzyme consisting of a catalyst and aptamer conjugate. (**B**) Schematic library of nucleoapzymes consisting of a catalyst linked directly, or through a space bridge, to the 3′- or 5′-ends of the aptamer scaffold, or where the catalyst separates the “split” aptamer subunits.

The selective binding properties of aptamers have been utilized to design hybrid nanostructures mimicking native enzymes-nucleoapzymes [7]. The covalent conjugation of a catalytic unit to sequence-specific aptamers provides means to duplicate the cooperative functions of the catalytic sites and binding sites in native enzymes, where the substrate acts as the ligand that binds to the aptamer. As the catalyst may be conjugated to the 3′-end, 5′-end or middle portions of the aptamer, or the catalytic unit may be linked to the 3′- or 5′-ends by different space elements, one may envisage the possibility to synthesize sets of catalytic-aptamer nucleoapzyme module exhibiting variable activities (Figure 12). Thus, it would be interesting to computationally elucidate the structure-catalytic function relationships with the set of nucleoapzymes and compare the computation features to the experimental results with the vision that in silico design of superior nucleoapzymes could be accomplished in the future.

The molecular engineering of the nucleoapzymes involves several stages. (i) One must define the catalytic transformation for which the nucleoapzymes should be designed. (ii) The availability of an aptamer for the reaction substrate is essential. This is elicited by the well-established SELEX procedure. (iii) A catalyst for the target chemical transformation should be selected. As many catalytic nucleic acid modules, DNAzymes, are available, they may be conjugated as a part of the nucleic acid sequence to yield the nucleoapzymes. Alternatively, a catalytic ligand, e.g., imidazole, or a transition metal complex catalyst, capable of catalyzing the target chemical transformation, should be covalently tethered to the aptamer sequence to yield the respective nucleoapzymes. The catalytic performance of the respective nucleoapzymes should then be compared to control systems that include the separated aptamer/catalytic site components and to a system comprising the catalytic site covalently linked to a nucleic acid composed of a scrambled, randomized sequence of the aptamer bases. In addition, the kinetic features of the nucleoapzymes should be analyzed by enzyme kinetic models, and the binding properties of the substrates to the respective nucleoapzyme structures should be evaluated, aiming to identify the structure/function relationships in the respective catalytic modules.

**Figure 13:**
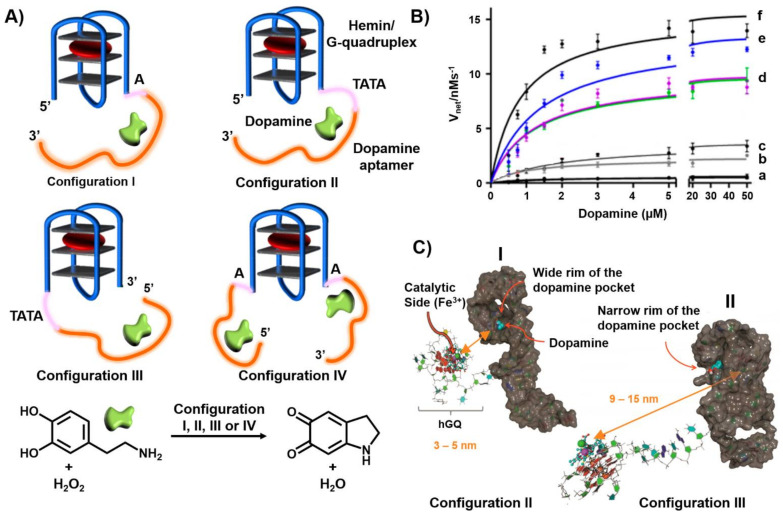
(**A**) Hemin/G-quadruplex-dopamine aptamer nucleoapzyme structures where in Configuration I the catalyst is linked to the 5′-end of the aptamer through a single A-bridge. In configuration II the catalyst is linked to the 5′-end of the aptamer through a TATA tether. In configuration III the catalyst is linked to the 3′-end of the aptamer through a TATA tether. In configuration IV the catalyst is linked to the 3′ and 5′-ends of the aptamer through a single A-bridge. The reaction driven by the systems correspond to the hemin/G-quadruplex catalyzed oxidation of dopamine by H_2_O_2_ to aminodopachrome. (**B**) Rates of oxidation of dopamine by H_2_O_2_ at different concentrations of dopamine: a = separated Hemin/G-quadruplex and dopamine aptamer; b = nucleoapzyme configuration III; c = scrambled sequence; d = nucleoapzyme configuration I; e = nucleoapzyme configuration II; f = nucleoapzyme configuration IV (**C**) Molecular dynamics energy minimized structures of: (I) nucleoapzyme in configuration II; (II) nucleoapzyme in configuration III. Reproduced with permission from ref. [172]. Copyright 2016 American Chemical Society.

In the past few years, our laboratory has developed the concept of nucleoapzymes, and two examples are introduced. The first example includes the conjugation of the hemin/G-quadruplex DNAzyme catalytic units to the dopamine aptamer to yield a series of nucleoapzymes catalyzing the H_2_O_2_-mediated oxidation of dopamine to dopachrome [172]. Four nucleoapzyme structures are exemplified in Figure 13A. The nucleoapzyme in configuration I includes the catalytic hemin G-quadruplex linked to the 5′-end of the aptamer through a single adenine (A) spacer. The nucleoapzyme in configuration II includes the hemin G-quadruplex conjugated to the 5′-end of the aptamer through a TATA spacer, and the nucleoapzyme in configuration III includes the hemin G-quadruplex linked to the 3′-end of the aptamer using the TATA spacer unit. In nucleoapzyme in configuration IV the hemin G-quadruplex is conjugated to the 5′-end and 3′-end of two aptamer sequences using single adenosine spacer units (A). Figure 13B depicts the kinetic features of the four configurations as compared to the separated hemin/G-quadruplex units, by providing the rates of dopachrome formation in the presence of variable concentrations of dopamine and an excess of the oxidizing agent H_2_O_2_. An additional control experiment includes a structure where the hemin/G-quadruplex catalyst is conjugated to the 5′-end of a nucleic acid composed of a scrambled, randomized sequence of the bases comprising the dopamine aptamer sequence (Figure 13B, curve (c)). The results demonstrated that all nucleoapzyme, structures I–IV, revealed substantial higher activities as compared to the separated hemin G-quadruplex and aptamer units. For the single hemin G-quadruplex-aptamer conjugates, the catalytic activity of the nucleoapzymes follows the order II > I > III. The nucleoapzyme in configuration II reveals a 20-fold enhanced catalytic activity compared to the separated units. All nucleoapzymes revealed a Michalis-Menten kinetic behavior and reached saturation kinetics consistent with the saturation of the aptamer binding sites by the dopamine substrate. Several conclusions were derived from these kinetic analyses and additional supporting experiments. (i) The nucleoapzyme in configuration II demonstrated superior catalytic properties as compared to configuration I and III (V_max_ = (13.5 ± 0.5) nm s^−1^; k_cat_/k_M_ = 14.1 × 10^−3^ s^−1^ mM. (ii) The hemin G-quadruplex linked to the scrambled sequence revealed a slight catalytic activity as compared to the separated units, attributed to the electrostatic attraction of the positively charged dopamine substrate to the negatively charged nucleic acid scaffold. (iii) The nucleoapzyme in configuration IV revealed superior catalytic properties due to the concentration of the reaction substrate by two aptamer sequences in the proximity of the catalytic site. (iv) The binding affinity of dopamine to the different nucleoapzymes, evaluated by fluorescence anisotropy measurements, revealed very similar dissociation constants implying that the differences in the catalytic performance of the nucleoapzymes did not originate from binding affinities to the aptamer receptors, suggesting that the different special orientation of the substrate in respect to the active catalytic sites provided the origin for the observed catalytic performance of the structures. To account for the structural features dictating the catalytic performance of the nucleoapzymes, we applied molecular dynamic (MD) simulations to probe the structural features of the nucleoapzymes [173,174,175,176] (Figure 13C). The MD simulations revealed that the hemin/G-quadruplex catalytic site is closer (3–9 nm) to the dopamine binding site in configuration II, as compared to the longer spatial separation of the catalytic site from the substrate binding site in configuration III (9–15 nm).

**Figure 14:**
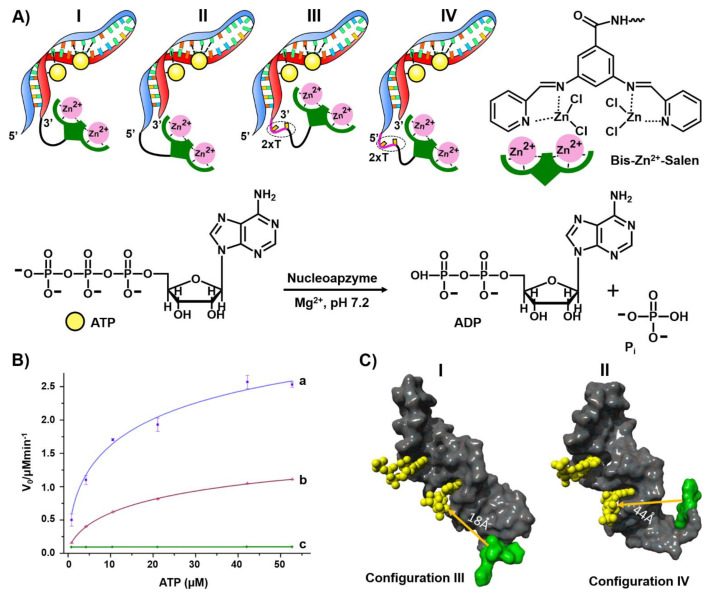
(**A**) Schematic configurations of bis-Zn^2+^-pyridyl salen modified ATP aptamers acting as nucleoapzymes for the catalyzed hydrolysis of ATP to ADP. (**B**) Rates of ATP hydrolysis to ADP by representative nucleoapzymes: a = nucleoapzyme in configuration III; b = nucleoapzyme in configuration IV; c = control experiment using the separated bis-Zn^2+^-pyridyl salen catalyst and the ATP aptamer. (**C**) Molecular dynamics energy-minimized structures of the nucleoapzyme in: Configuration III (I) and in Configuration IV (II). Reproduced with permission from ref. [177]. Copyright 2020 Wiley-VCH.

The second example for constructing nucleoapzymes includes the modification of the aptamer with a transition metal complex as catalytic unit. This is introduced by describing a set of nucleoapzymes mimicking the catalytic activity of ATPase; the hydrolysis of ATP to ADP [177]. A set of nucleoapzymes consisting of the ATP aptamer modified with the bis-Zn^2+^ pyridyl-salene complex was synthesized, (Figure 14). This included the linkage of the Zn^2+^-pyridyl-salene complex directly to the 3′- and 5′-ends of the ATP-aptamer, (configuration I and II), and the nucleoapzymes composed of the bis- Zn^2+^-pyridyl-salene complex linked to the 3′- and 5′-ends of the ATP aptamer using a two thymine spacer (2× T), (configurations III and IV, respectively). Figure 14B depicts the kinetic features corresponding to the hydrolysis of ATP to ADP by the nucleoapzyme III, curve (a) and nucleoapzyme IV, curve (b). While no hydrolysis of ATP could be detected in the presence of the separated bis-Zn^2+^-pyridyl-salene complex and ATP aptamer, the catalytic properties of all nucleoapzymes were demonstrated, following the order III > I > IV > II. For example, for nucleoapzyme in configuration III, k_cat_ = 688 × 10^−2^ min^−1^ and K_M_ = 38 ± 7 μM, whereas for nucleoapzyme in configuration IV, k_cat_ = 297 × 10^−2^ min^−1^ K_M_ = 33 ± 6 μM. As the binding affinities of ATP to the different nucleoapzymes were similar, K_d_ = 19 μM, it was suggested that the spatial positioning of ATP in the aptamer receptor scaffold, with respect to the catalyst, is favored in nucleoapzyme III compared to nucleoapzyme IV. Indeed, MD simulations indicated that the catalytic site in the nucleoapzyme in configuration III is positioned in a sterically favored configuration with respect to the hydrolytic reaction site, compared to the spatial separation of the catalytic site from the reaction site in the nucleoapzyme in configuration I (Figure 14). While the distance separating the catalytic site from the reaction site in configuration III is 18 Å, the distance in configuration IV is 44 Å. The concept of nucleoapzymes was further extended to include Cu(II)- or Fe(III)-terpyridine-functionalized dopamine aptamer for the catalyzed H_2_O_2_-mediated oxidation of dopamine [178] and the Fe(III)-terpyridine-modified tyrosinamide aptamer for the oxidative oxygen insertion into tyrosinamide [65]. The functionalization of the cholic acid aptamer with the imidazole ligand led to the catalyzed hydrolysis of a cholic acid ester substrate [179].

## 5. DNA Motor Systems Driven by Aptamer-Ligand Complexes

The development of DNA motor systems is a major accomplishment in the area of DNA nanotechnology. The assembly of signal-triggered reconfigurable DNA nanostructures is the background concept behind the construction of mechanically-driven DNA devices [180,181]. Different mechanically reconfigured nanostructures performing machine-like functions operating as tweezers [182,183,184], walkers [185,186] or pendulums [187] were reported, and the mechanical reversible reconfiguration of nanostructures such as origami tiles [188], interlocked DNA rings in catenane [189,190,191,192] or rotaxane [193,194,195] configurations and, the reversible opening and closure of DNA cages [151,196] by auxiliary triggers were accomplished. Different applications of these DNA machineries were suggested including their use as switchable catalysts [197], logic gates [198,199,200], plasmonic devices [201] and drug carriers [202,203].

**Figure 15:**
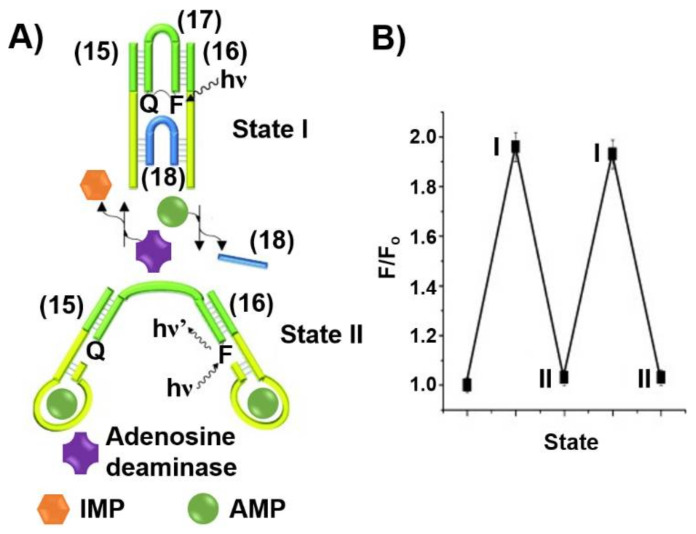
(**A**) An AMP/Adenosine deaminase triggered reversible opening of a DNA tweezer machine. The formation of the AMP/AMP aptamer complexes with the tweezer “arms” open the tweezer while the adenosine deaminase catalyzed conversion of AMP to inosine monophosphate (IMP) separates the AMP/AMP aptamer complexes and restores the closed tweezer. The opening and closure of the tweezer is recorded by following the fluorescence of the Cy5 fluorophore, that is the spatial separation from the quencher unit controls the fluorescence intensity transduced by the fluorophore. (**B**) Cyclic fluorescence changes transduced from the nanodevice upon opening (I) and closure (II). Reproduced with permission from ref. [204]. Copyright 2009 Wiley-VCH.

The triggered formation and dissociation of aptamer-ligand complexes provides a general means to construct DNA machine-like devices. This is exemplified in Figure 15A with the assembly of a DNA tweezer triggered by ligand-aptamer complexes into “open” and “closed” configurations [204]. The tweezer in the closed state I is composed of two arms (**15**) and (**16**) interlocked by two bridging strands, (**17**) and (**18**), that hybridize with counter complementary domains associated with the arms (**15**) and (**16**). The arms (**15**) and (**16**) include sequence-specific domains corresponding to the adenosine monophosphate aptamer, AMP aptamer. In the presence of AMP, the formation of the AMP ligand/AMP aptamer complexes releases the strand (**18**), resulting in the formation of the open tweezer configuration, state II. Subjecting the open tweezer to the enzyme adenosine deaminase transforms the AMP ligand to inosine monophosphate (IMP) that lacks affinity towards the AMP aptamer. This results in the rehybridization of the free strand (**18**) with the open tweezer to regenerate the closed tweezer state, state I. By the labeling of the inner inter-bridging strand (**17**) with a fluorophore-quencher pair, the dynamic opening and closure of the tweezers is followed spectroscopically. In the closed state of the tweezer, state I, the spatial proximity between the fluorophore-quencher units led to the effective quenching of the fluorophore. In turn, the AMP-guided separation of the tweezers through the formation of AMP/AMP aptamer complexes, state II, results in the spatial separation between the fluorophore-quencher units and the recovery of the fluorescence of the fluorophore-probe. By the cyclic treatment of the tweezer with AMP and adenosine deaminase, the dynamic mechanical switching of the tweezer between the closed and open states was demonstrated, Figure 15B.

**Figure 16:**
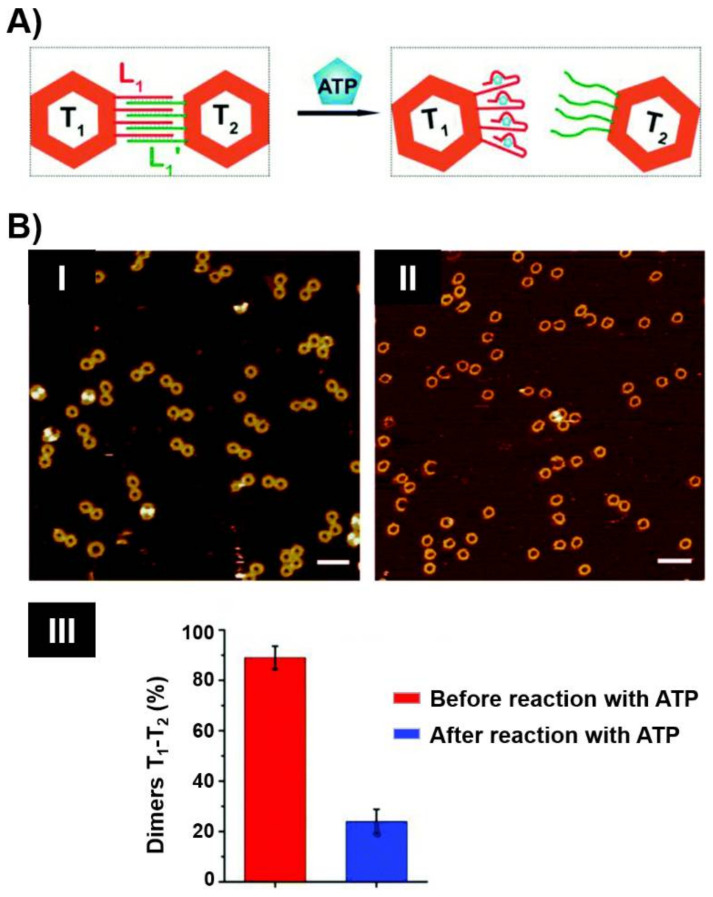
(**A**) ATP-triggered separation of an origami dimer by the separation of interdimer bridging units through the formation of ATP/ATP aptamer complexes. (**B**) Atomic force microscopy (AFM) images of: Panel I—the dimer origami structures; Panel II—the ATP-driven separated origami tiles: Panel III—statistical analysis of the origami tiles generated upon treatment of the origami dimers with ATP. Reproduced with permission from ref. [216]. Copyright 2017 Royal Society of Chemistry.

The assembly of origami nanostructures by the programmed interaction of a long single-strand DNA, e.g., M13 phage, with hundreds of computer-designed “staple” oligonucleotide strands to yield 2D or 3D shapes has become a common practice in DNA nanotechnology. By the conjugation of nucleic acid tethers to the staple units comprising the origami scaffolds or the edges of the origami tiles, functional units for the successive modification of the origami structures were demonstrated [205,206]. Programmed attachment of nanoparticles [207,208], proteins [209] or polymers [210,211] to the protruding tethers associated with the origami structures was realized. These versatile functionalities of DNA origami nanostructures were applied to develop nanostructured aptamer-based origami switches and machines [212,213,214,215]. Figure 16A depicts the separation of two interlinked origami frames T_1_ and T_2_ through the formation of aptamer-ligand complexes [216]. The bridging of the origami frames by L_1_/L_1′_ duplex, where L_1_ includes the aptamer sequence against ATP, provided the binding motif to assemble the dimer origami structure. In the presence of ATP, the formation of the ATP/ATP aptamer complexes led to the separation of the dimers. The dimer to monomer transitions were followed by atomic force microscopy, AFM (Figure 16B). 

**Figure 17:**
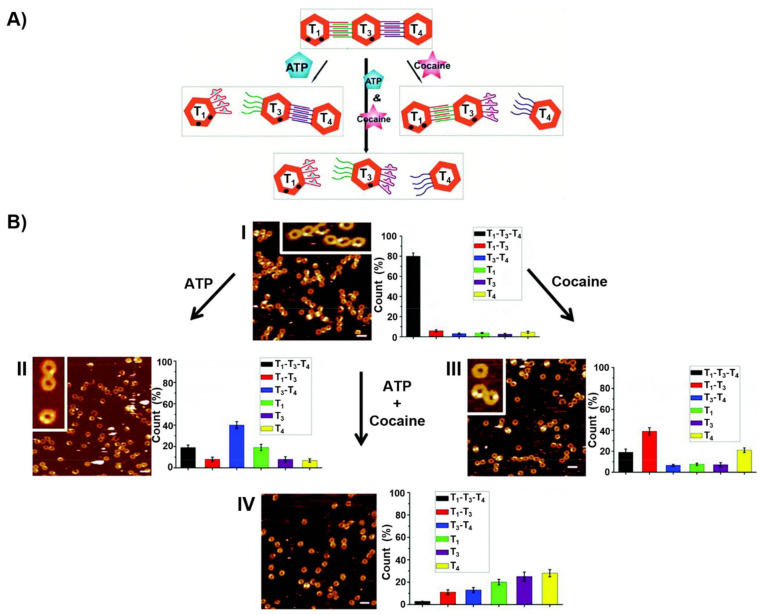
(**A**) Dictated triggered separation of an origami trimer using different aptamer interconnecting bridging units. Treatment of the origami trimer T_1_–T_3_–T_4_ with ATP yield the separation of tile T_1_ through the formation of the ATP/ATP aptamer complex and the intact T_3_–T_4_ dimer. Subjecting the trimer to cocaine yield the T_1–_T_3_ dimer and separated tile T_4_ through the formation of the cocaine/cocaine aptamer complex. Treatment of the trimer with cocaine and ATP led to the concomitant separation of the trimer to the monomer tiles T_1_, T_3_, T_4_. (**B**) AFM images corresponding to: Panel I—the origami trimer T_1_–T_3_–T_4_; Panel II—the ATP triggered separation of the trimer T_1_–T_3_–T_4_ into the monomer tile T_1_ and the dimer tile T_3_–T_4_; Panel III—the cocaine triggered separation of the trimer T_1_–T_3_–T_4_ into the dimer tile T_1_–T_3_ and the monomer tile T_4_; Panel IV—the cocaine and ATP triggered separation of the trimer T_1_–T_3_–T_4_ into the monomer tiles T_1_, T_3_, T_4_. The AFMimages are accompanied by statistical analysis of the percentage of the tiles. Reproduced with permission from ref. [216]. Copyright 2017 Royal Society of Chemistry.

In addition, edge-crosslinked origami frames trimers, T_1_–T_3_–T_4_, were designed by the programmed modification of counter edges of the origami tile T_3_ with the strands L_1′_ and L_2_ and the subsequent hybridization of the L_1_-modified tile T_1_ and of the L_2′_-functionalized tile T_4_ to yield the respective duplex bridged origami trimer T_1_–T_3_–T_4_. The strands L_1_ and L_2_ correspond to the ATP and the cocaine aptamer, respectively (Figure 17A) [216]. In the presence of ATP, the origami frame could be separated, leading to the monomer T_1_ and the dimer T_3_–T_4_. In the presence of cocaine, the trimer structure could be separated through the formation of the cocaine/cocaine aptamer complexes, leading to the formation of the T_1_–T_3_ dimer and the T_4_ monomer. In the presence of ATP and cocaine the trimer structure could be separated into individual origami frames, T_1_, T_3_ and T_4_ through the formation of the respective ATP/ATP aptamer complexes and cocaine/cocaine aptamer complexes. The specific frame structures could be identified by labelling of the frame T_1_ with two nucleic acid hairpin markers (presented as dots) and labelling of the frame T_3_ with a single hairpin marker (presented as a dot). The programmed dictated separation of the origami trimer by ATP, cocaine, and a mixture of ATP and cocaine could then be imaged by AFM and the quantitative analysis of the respective monomer/dimer compositions in the respective scan areas was also accomplished (Figure 17B).

**Figure 18:**
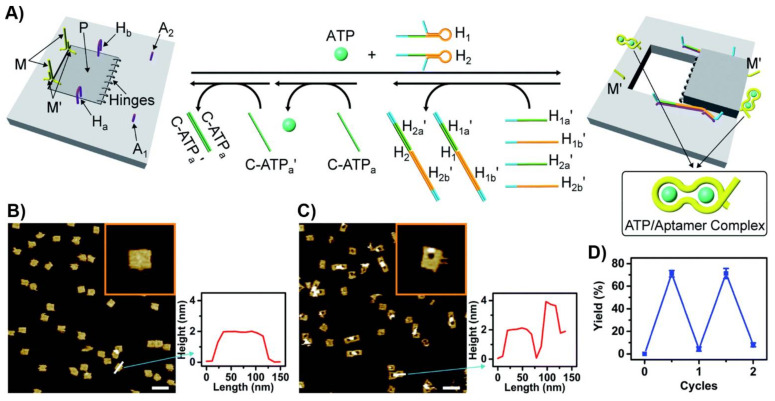
(**A**) Schematic “mechanical” open of a “window patch” in an origami tile using the formation of ATP/APT aptamer complexes as a mechanism to unlock the “window”. The formation of the ATP/ATP aptamer complexes followed by the binding of helper strands to “window” handles and their stretching to bind the stature foothold, associated with the origami scaffold led to the fixed rigid opening of the “window” and to the formation of a nanocavity in the origami raft. The reverse unlocking of the foothold binding strand and the release of ATP relocks the caped “window” structure. (**B**) AFM images of the locked origami structures and accompanying cross-section analysis. (**C**) AFM image of the cavity containing unlocked origami rafts and accompanying cross-section analysis. (**D**) Switchable and reversible ATP-driven opening and closure of nanocavities in the origami raft. Reproduced with permission from ref. [217]. Copyright 2020 Royal Society of Chemistry.

A nanoengineered DNA-origami raft was designed as a functional nanostructure for the “mechanical” switchable opening and closure of a “window” in the raft (Figure 18A) [217]. The origami tile was engineered to include a subdomain (window) linked to the raft by eight hinges, two hairpin “arms”, H_a_ and H_b_, and two duplex locks M/M’ composed of two complementary strands M and M’, where M includes the ATP sequence. In addition, the origami scaffold includes two additional protruding tethers, A_1_ and A_2_, acting as capturing strands. In the presence of ATP and the added two hairpin structures, H_1_ and H_2_, the two duplexes M/M’ are unlocked and the opening of the hairpins H_1_ and H_2_ by hybridization with the arms H_a_ and H_b_ yield “ropes” that pool and open the window in the origami raft through hybridization (“binding”) of the ropes with the anchor strands A_1_ and A_2_. The reverse release of the “helper” rope strand with the strands H_1a_’, H_1b_’, H_2a_’ and H_2b_’ and the release of the ATP ligand by the counter ATP_a_’ aptamer strand, followed by washing of the resulting ATP-ATP_a_’ aptamer complexes, uncage the strands M and M’ allowing the regeneration of the locked “window” origami. The unlocking of the closed origami tile configuration and the ATP-driven open “window” configuration of the nanocavity were imaged by AFM microscopy. The resulting AFM images, and the respective cross-section analyses of the respective tiles, are shown in Figure 18B,C. The reversible mechanical opening and closure of the cavities is demonstrated in Figure 18D. 

**Figure 19:**
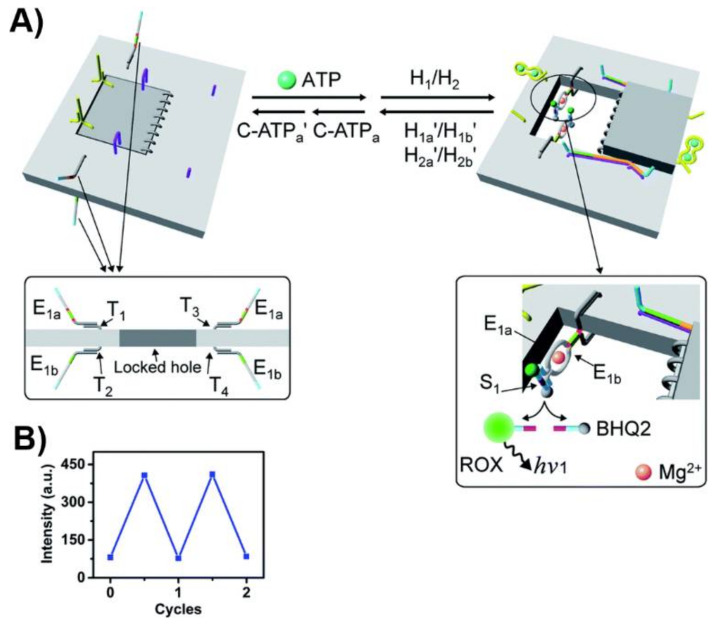
(**A**) ATP-driven opening and closure of a “window” in an origami scaffold and the triggered “ON”/”OFF” activation of the Mg^2+^-ion-dependent DNAzyme in the structurally confined cavity. Two subunits of the DNAzyme are positioned at the top and below the origami scaffold. Opening of the “window” leads to the self-assembly of the Mg^2+-^ion-dependent DNAzyme in the cavity resulting in the activation of the DNAzyme and the cleavage of the fluorophore/quencher-modified substrate. The fluorescence of the fragmented product transduces the activity of the DNAzyme. The closure of the “window” separates the two DNAzyme subunits leading to the switched off state of the DNAzyme. (**B**) Cyclic and switchable activation of the Mg^2+^-ion dependent DNAzyme in the cavity upon the switchable opening/closure of “window”. Reproduced with permission from ref. [217]. Copyright 2020 Royal Society of Chemistry.

In addition to the mechanical unlocking and closure of the nanocavities in the origami tiles, switchable “ON” and “OFF” catalysis in the confined nanoholes generated in the origami tiles was demonstrated. Towards this goal, the origami tile at the boundary of the “window” domain was functionalized on the upper face with two protruding tethers, T_1_ and T_3_, and from the bottom face with two additional protruding tethers, T_2_ and T_4_. The strand E_1a_, E_1b_, being subunits of the Mg^2+^-ion-dependent DNAzyme, were hybridized to the tethers T_1_/T_3_ and T_2_/T_4_ on the opposite faces of the origami tile. In the locked configuration of the tiles, the communication between the two subunits on the opposite faces of the origami tile is prohibited. The mechanical unlocking of the “window”allowed, however, the assembly of two Mg^2+^-ion-dependent DNAzyme structures in the confined cavity of the nanostructure. The hydrolytic catalyzed cleavage of the ROX/BHQ-functionalized ribonucleobase substrate of the DNAzyme units, resulted in the ROX fluorescence of the fragmented product generated by the supramolecular DNAzyme units (Figure 19). By the cyclic opening and closure of the “window” upon treatment of the system with ATP and its relocking by removal of the ATP, the catalytic process in the cavities was switched reversibly across “ON” and “OFF” states, Figure 19B.

## 6. Conclusions

The specific recognition properties of aptamers have found broad applications in chemistry, medicine and materials science. Numerous applications of aptamers in analytical chemistry, e.g., sensing and separation, biomedical applications such as imaging, therapeutics and tissue targeting and material science, reflected by the development of new catalysts and the synthesis of soft materials, have been demonstrated in the past two decades. The integration of aptamers with nanoparticles, or the integration of aptamers with DNA or proteins, introduced important aptamer-nanoparticle hybrids and aptamer nanostructures, thus advancing the field of nanotechnology and nanobiotechnology. These aptamer-nanoparticle hybrids, or programmed aptamer nanostructures, combine the unique selective recognition and targeting properties of the aptamers with unique optical, electronic or catalytic properties of nanoparticles, or enabled the integration of aptamers with soft nanostructures, such as DNA tetrahedra or DNA origami, to yield functional materials for broad utility in the fields of sensing, imaging, therapy, drug delivery and more. The present perspective introduced some activities originated from our laboratory, demonstrating the applications of aptamer-functionalized hybrid structures for sensing, drug delivery, catalysis and mechanical features. Despite the progress in the field, one might identify future challenges. (i) Recent activities demonstrated the significance of molecular dynamic simulations to understand, and improve by base mutations, the ligand-aptamer interactions. Such molecular simulations followed by experimental validation could introduce superior aptamers. (ii)The chemical modification of aptamers by redox or photochemical functionalities led to aptamers of switchable binding properties and even enhanced binding properties. The introduction of new chemical functions into aptamer scaffolds is anticipated to lead to new applications of aptamers for sensing and molecular machine design. (iii) The functionalization of nanoparticles and nanomaterials with aptamers has been applied, until now, mainly to develop carriers for drug delivery and imaging. The recent extensive efforts to apply nanoparticles as catalytic “nanozymes” suggest that the functionalization of these particles with aptamers could yield improved “aptonanozymes”, i.e., catalytic hybrids that concentrate the reaction substrate at the catalytic site, in analogy to native enzymes. In summary, the broad applications of aptamers demonstrated in the past two decades promise exciting future developments in chemistry, nanomedicine and material science.

## Data Availability

Not applicable.
